# #DeOlhoNosCorais: a polygonal annotated dataset to optimize coral monitoring

**DOI:** 10.7717/peerj.16219

**Published:** 2023-11-06

**Authors:** Daniel P. Furtado, Edson A. Vieira, Wildna Fernandes Nascimento, Kelly Y. Inagaki, Jessica Bleuel, Marco Antonio Zanata Alves, Guilherme O. Longo, Luiz S. Oliveira

**Affiliations:** 1 Department of Informatics, Federal University of Paraná, Curitiba, PR, Brazil; 2 Department of Oceanography and Limnology, Federal University of Rio Grande do Norte, Natal, RN, Brazil

**Keywords:** Convolutional neural network, Machine learning, Computer vision, Marine ecology

## Abstract

Corals are colonial animals within the Phylum Cnidaria that form coral reefs, playing a significant role in marine environments by providing habitat for fish, mollusks, crustaceans, sponges, algae, and other organisms. Global climate changes are causing more intense and frequent thermal stress events, leading to corals losing their color due to the disruption of a symbiotic relationship with photosynthetic endosymbionts. Given the importance of corals to the marine environment, monitoring coral reefs is critical to understanding their response to anthropogenic impacts. Most coral monitoring activities involve underwater photographs, which can be costly to generate on large spatial scales and require processing and analysis that may be time-consuming. The Marine Ecology Laboratory (LECOM) at the Federal University of Rio Grande do Norte (UFRN) developed the project “#DeOlhoNosCorais” which encourages users to post photos of coral reefs on their social media (Instagram) using this hashtag, enabling people without previous scientific training to contribute to coral monitoring. The laboratory team identifies the species and gathers information on coral health along the Brazilian coast by analyzing each picture posted on social media. To optimize this process, we conducted baseline experiments for image classification and semantic segmentation. We analyzed the classification results of three different machine learning models using the Local Interpretable Model-agnostic Explanations (LIME) algorithm. The best results were achieved by combining EfficientNet for feature extraction and Logistic Regression for classification. Regarding semantic segmentation, the U-Net Pix2Pix model produced a pixel-level accuracy of 86%. Our results indicate that this tool can enhance image selection for coral monitoring purposes and open several perspectives for improving classification performance. Furthermore, our findings can be expanded by incorporating other datasets to create a tool that streamlines the time and cost associated with analyzing coral reef images across various regions.

## Introduction

**Figure 1:**
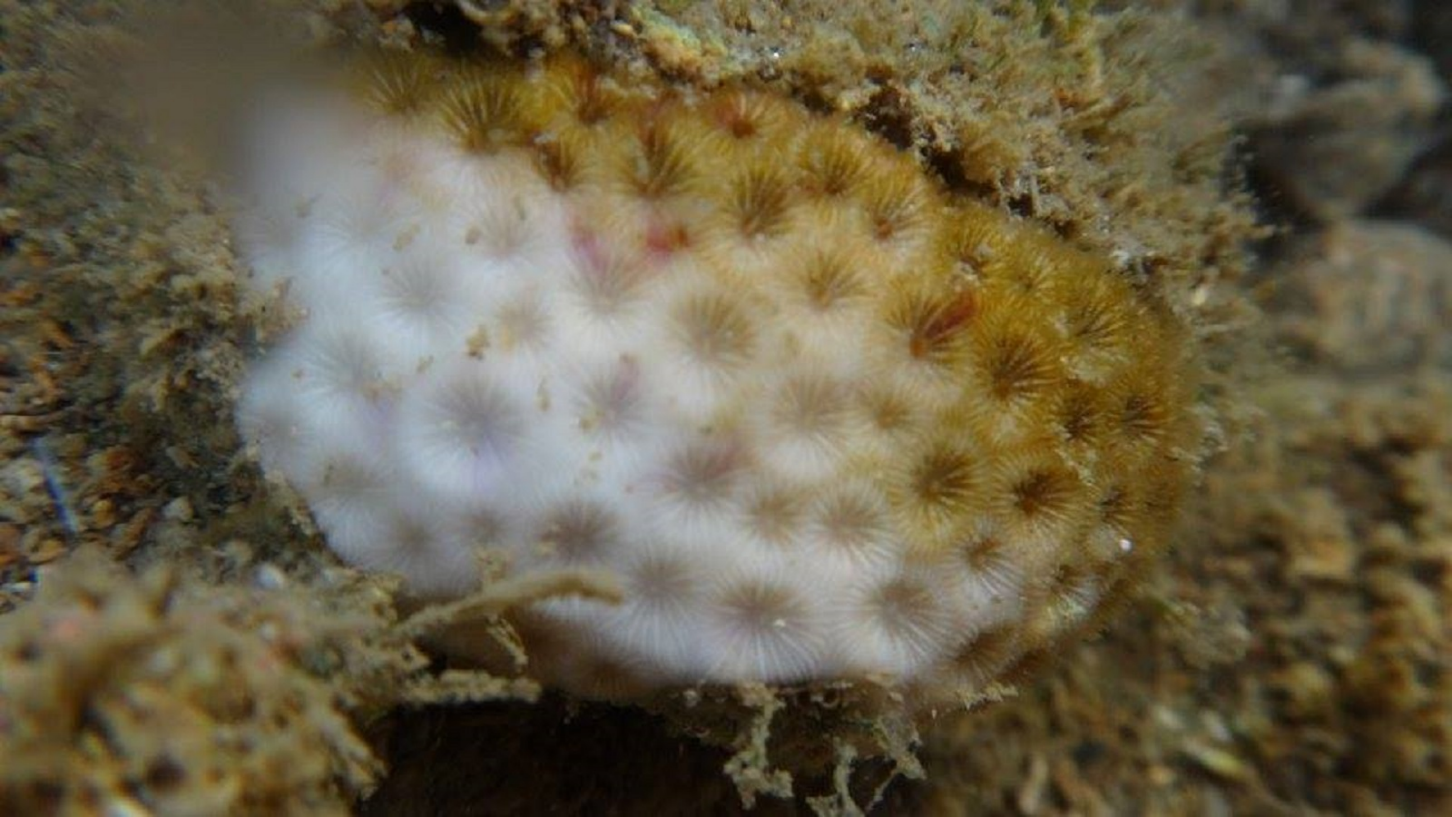
Example of a bleaching event for a coral of the species *Siderastrea stellata* (Furtado, 2022).

Corals are cnidarians that live in colonies and produce calcium carbonate skeletons, which build coral reefs providing habitat for various species including algae, fungi, bacteria, and fish. These reefs sustain approximately 25% of all marine species (Chen et al., 2015). Corals also hold significant economic importance for coastal regions, contributing about US$ 30 billion annually through fishing and tourism linked to the marine ecosystem. Furthermore, they supply food and resources to approximately 500 million people worldwide (Hoegh-Guldberg, 2011). However, due to climate change, coral coverage is shrinking at an alarming rate of 1–2% per year (Hoegh-Guldberg, 2011), driven by ocean warming that prompts corals to expel their photosynthetic endosymbionts, a phenomenon known as coral bleaching (Hoegh-Guldberg, 1999). This event leads to the white appearance (Fig. 1), resulting from a decline in endosymbiont abundance, which highlights the white coral skeleton through its gelatinous and transparent tissue. While corals can generally adapt to temperature fluctuations in the ocean, frequent and severe warming events often lead to extensive coral mortality (Hughes et al., 2018).

Given the paramount importance of coral reefs to the marine ecosystem, monitoring becomes essential to mitigate potential environmental and economic impacts. Currently, coral monitoring primarily relies on images captured by divers. In areas where diving is restricted, an Autonomous Underwater Vehicle (AUV) can serve as an alternative. Modasshir et al. (2018) demonstrated an AUV equipped with a Convolutional Neural Network (CNN) designed to detect and identify various coral species. The tracking mechanism also provides a total count for each species per transect. Marine ecology experts store and analyze these images to identify any anomalies that require thorough processing. Several researchers have been embracing machine learning techniques to streamline image analysis. As of our current knowledge, the seminal work was presented in 2012 by Beijbom and colleagues (Beijbom et al., 2012). They introduced the Moorea Labeled Corals (MLC) dataset along with a handcrafted feature set based on texture and color. In 2013, Shihavuddin et al. (2013) employed the Grey-Level Co-occurrence matrix (GLCM) to train diverse classifiers for coral classification. Nevertheless, the recent achievements of deep learning (DL) techniques in various computer vision applications have prompted researchers to reevaluate the coral classification problem.

In this context, it is worth mentioning three related works published by Mahmood and his colleagues. In Mahmood et al. (2016), they combined handcrafted representations with VGG (Simonyan & Zisserman, 2014) features to distinguish between live corals and bleached ones. More recently, they evaluated the effectiveness of deep residual networks (ResNets) (Mahmood et al., 2020) and transfer learning (Ospina et al., 2020). They concluded that DL significantly outperforms traditional machine learning techniques. These findings were further supported by Gómez-Ríos et al. (2019) and Lumini, Nanni & Maguolo (2019), who achieved superior results compared to Shihavuddin et al. (2013) on the RSMAS and EILAT datasets (Shihavuddin, 2017) using DL.

A recent milestone in classification is CoralNet (Chen et al., 2021), a website that provides tools for manual, semi-automatic, and automatic analysis of coral reef images. As of 2021, the website contained 1,741,855 images from 2,040 distinct sources, with over 65 million annotations uploaded by nearly 3,000 users. The machine learning engine, CoralNet 1.0, employs EfficientNet-B0 (Tan & Le, 2019) as a feature extractor and a Multi-layer Perceptron as a classifier.

Raphael et al. (2020) demonstrated the effectiveness of automated DL classification in optimizing coral monitoring in a shallow reef in the Gulf of Eilat compared to manual classification. They retrieved 5,000 photographic images from video samples filmed between June 2017 and June 2018, facing challenges such as image quality, distance from the object, angle of view, and lighting conditions. They reported an accuracy of 80.13% using ResNet-50 (He et al., 2015).

Another field of study that has benefited from the advances in DL is semantic image segmentation (Zhu et al., 2016). This kind of algorithm aims to assign a category label to each image pixel. However, these algorithms require good quality densely labeled segmentation data to perform well. Since such data is rarely available, Alonso et al. (2017) proposed a pipeline to generate synthetic segmentation data. The authors argue that their method can yield high-quality segmentation. A comparison of DL methods for the semantic segmentation of coral reef images can be found in King, Bhandarkar & Hopkinson (2018).

As far as our knowledge extends, the Mosaics UCSD dataset (Edwards et al., 2017) stands as the only dataset featuring densely labeled images. It encompasses 16 mosaics with high resolution and 1,290 M labeled pixels distributed across 35 semantic classes. Building on their prior work, Alonso et al. (2019) aimed to create a densely labeled dataset from a sparsely labeled one, and they carried out their initial experiment using the Mosaics UCSD dataset. The authors cropped the images into 512 × 512 segments, resulting in 4,957 images. They employed a modified architecture of Deeplabv3 (Chen et al., 2017) and achieved a mIoU of 51.57% in their experiments on the dataset.

**Figure 2:**
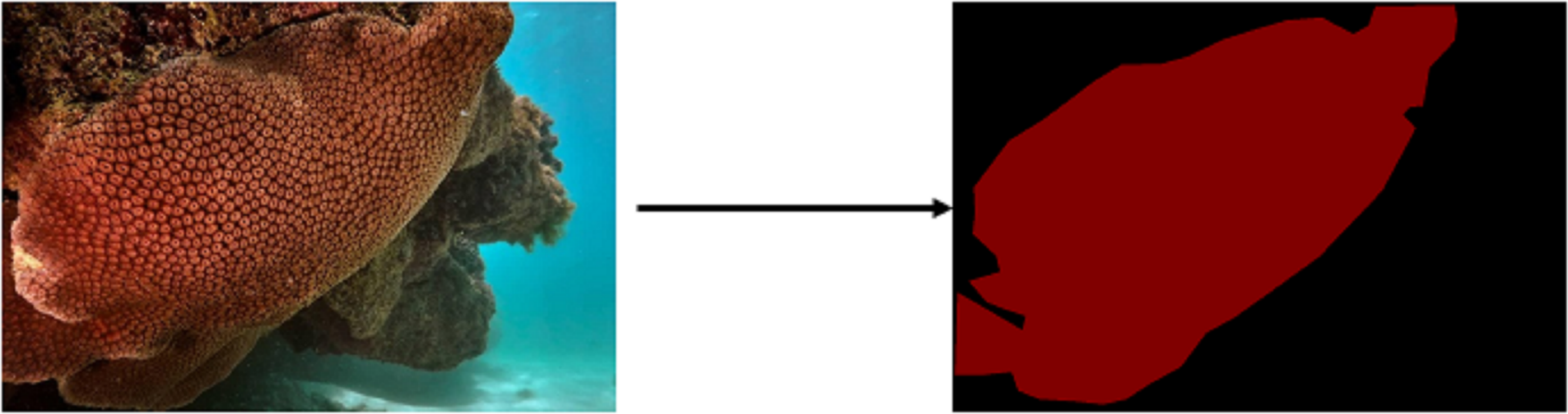
Example of the segmentation map for an image which contains a coral of the species *Montastraea cavernosa* (Furtado, 2022).

In this study, we introduce the “#DeOlhoNosCorais” dataset, comprising 1,411 images showcasing 21 classes of corals that include scleractinian corals, hydrocorals and zoanthids along the Brazilian coast, accompanied by their respective segmentation maps. For the analytical procedures we treated scleractinian corals, hydrocorals and zoanthids simply as corals. The dataset encompasses a total of 7,082 M labeled pixels, which is 5.5 times larger than the Mosaics UCSD dataset. This segmentation information significantly contributes to the exploration of new semantic segmentation strategies for coral images. In comparison to other datasets like MLC and Pacific Labeled Corals (PLC), our proposed dataset is a substantial advancement. The dataset is publicly accessible (https://doi.org/10.5281/zenodo.7338208). The segmentation map, as illustrated in Fig. 2, provides a comprehensive depiction of the coral’s location within the image.

“#DeOlhoNosCorais” is an initiative led by the Marine Ecology Laboratory at the Federal University of Rio Grande do Norte (UFRN), Brazil, aimed at advancing the monitoring of coral health along the Brazilian coastline. The core objective of this project is to encourage users of various social media platforms to share photographs of coral reefs that they have captured using their personal equipment. These images are to be accompanied by the designated project hashtag (#DeOlhoNosCorais). Through the utilization of this collaborative approach, contributions from individuals across social media channels contribute to the dynamic expansion of the existing dataset. Contributions are not restricted to corals, as citizen scientists often contribute with pictures of hydrocorals and zoanthids.

**Figure 3:**
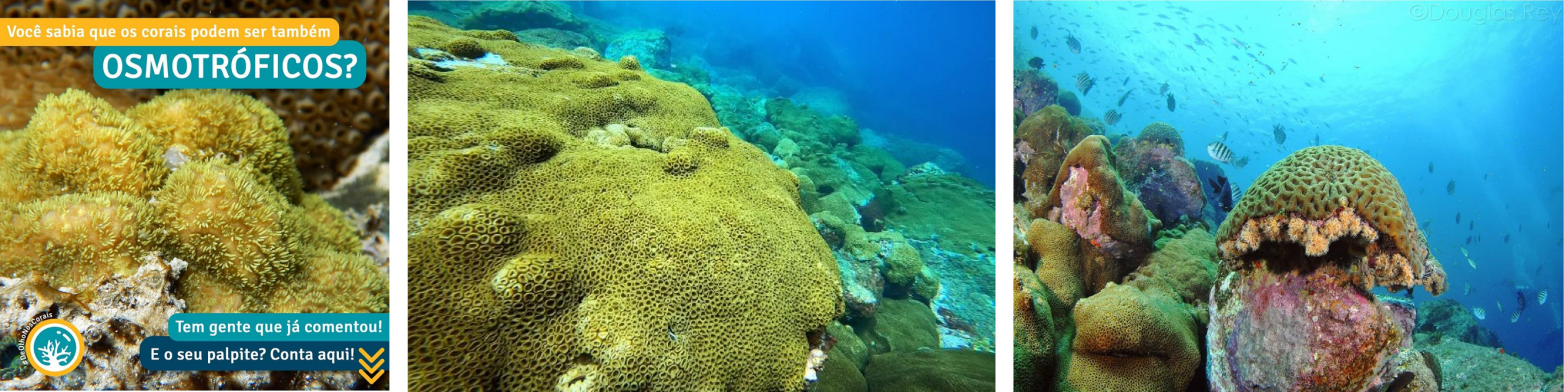
Some challenging images from the #DeOlhoNosCorais dataset. Text: Did you know that corals can also be OSMOTROPHIC? Some people have already commented! What’s your guess? Share it here! (Furtado, 2022).

Simultaneously, this dataset can serve as training data for machine learning models capable of classifying shared photographs in real-time. Furthermore, the development of a semantic segmentation model allows for precise isolation of corals within these images. Despite its inherent advantages, the dataset presents several challenges for machine learning algorithms. These challenges encompass the presence of noise elements, as exemplified in Fig. 3, such as textual overlays and watermarks, as well as fluctuations in color patterns leading to variations in saturation, white balance, and brightness. Inconsistencies in image resolutions also exist.

Drawing parallels with the achievements showcased by the CoralNet project (Chen et al., 2021), these models bear significant potential for expediting the classification and segmentation of numerous photographs captured by divers during their underwater expeditions, as highlighted by Raphael et al. (2020). González-Rivero et al. (2020) concluded in their article that CNNs brought about enhanced image classification methods through automated image recognition. This method outperformed traditional machine learning approaches, demonstrating a reliable concurrence of 97% between expert and automated assessments, alongside an overall error rate of 4%. Analyses of community composition indices underscored the persistence of this agreement across various bioregions, maintaining levels of 83% to 94%. While error rates differed among taxonomic groups, they indicated that AI-powered systems could achieve a functional taxonomic resolution similar to that of trained human observers. By implementing artificial intelligence for automated image classification, the time-consuming data processing and reporting constraints in coral reef monitoring can be drastically mitigated. This technology accelerates image analysis by at least a factor of 200 and incurs only a fraction of the expenses associated with manual image annotation (1%).

To establish a baseline for future comparisons, we evaluated this dataset in two distinct scenarios: classification and segmentation. For classification, we conducted two experiments: using the full image and using sub-images. Following the findings reported by Mahmood et al. (2020), we employed two deep learning strategies. We also applied the Local Interpretable Model-Agnostic Explanations (LIME) algorithm to interpret the classification results. In terms of segmentation, we present results for the binary semantic segmentation approach using U-Net.

## Dataset

The initial release of the #DeOlhoNosCorais dataset encompasses 1,411 images with 21 classes. These images contained scleractinian corals, hydrocorals, and zoanthids but are simply referred to as corals in the analytical procedures, results, and discussion. The images were captured between 2014 and 2021 in Brazilian reefs. In addition to the publicly available images collected from Instagram, the dataset includes additional images contributed by divers from the LECOM-UFRN research group. The dataset were collected as previously described in Furtado (2022).

**Figure 4:**
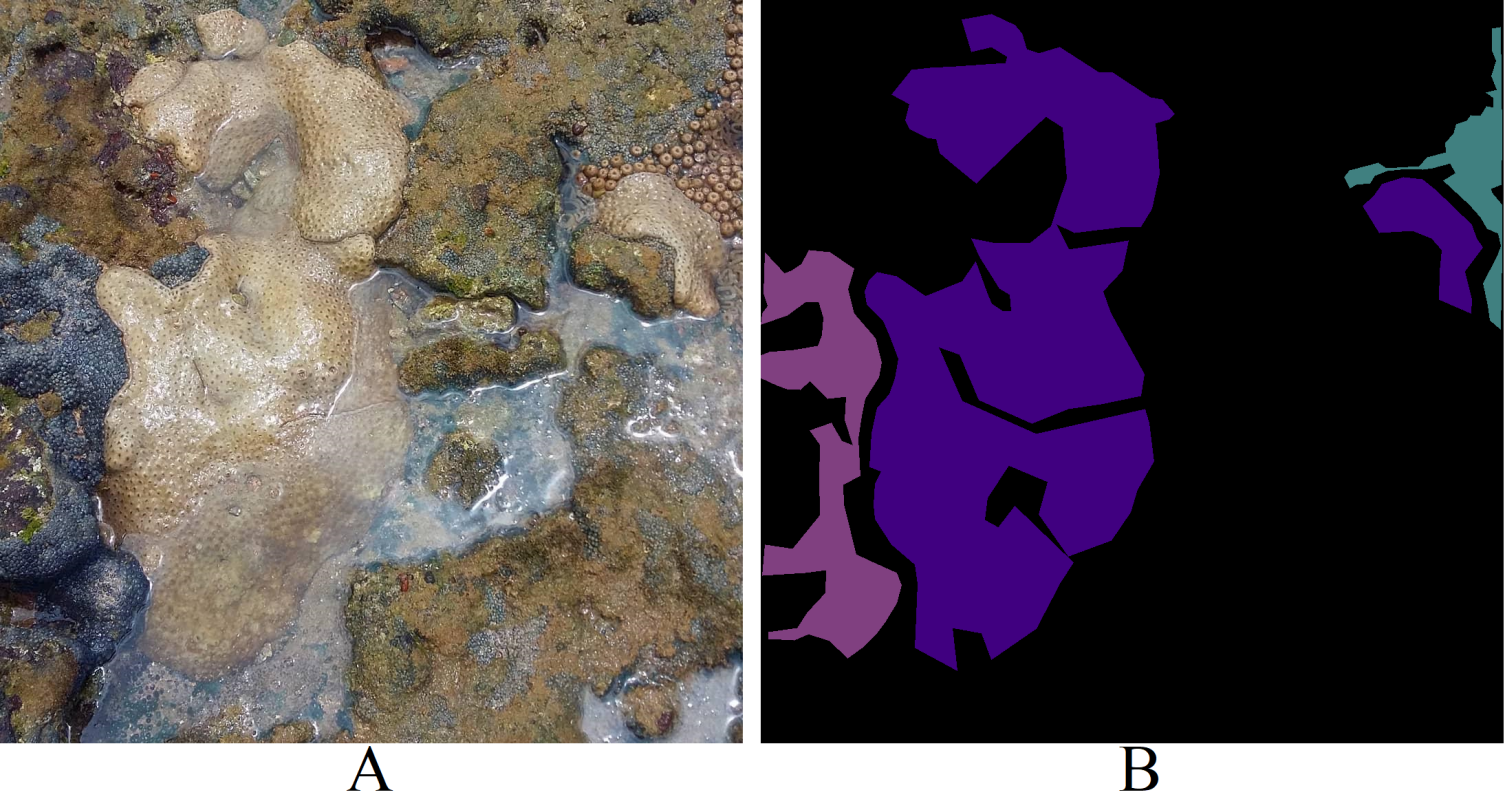
Dataset labeling (A) original image - major class: *Palythoa caribaeorum* (B) segmentation map - pink: *Zoanthus sociatus*, purple: *Palythoa caribaeorum*, blue: *Palythoa* spp (Furtado, 2022).

Each image within the dataset possesses two labels: its primary class (Fig. 4A) and the corresponding segmentation map (Fig. 4B). LECOM-UFRN labeled the images using the Labelme tool (Wada, 2016), and duplicate images were eliminated using the FiftyOne tool (Moore & Corso, 2020).

**Figure 5:**
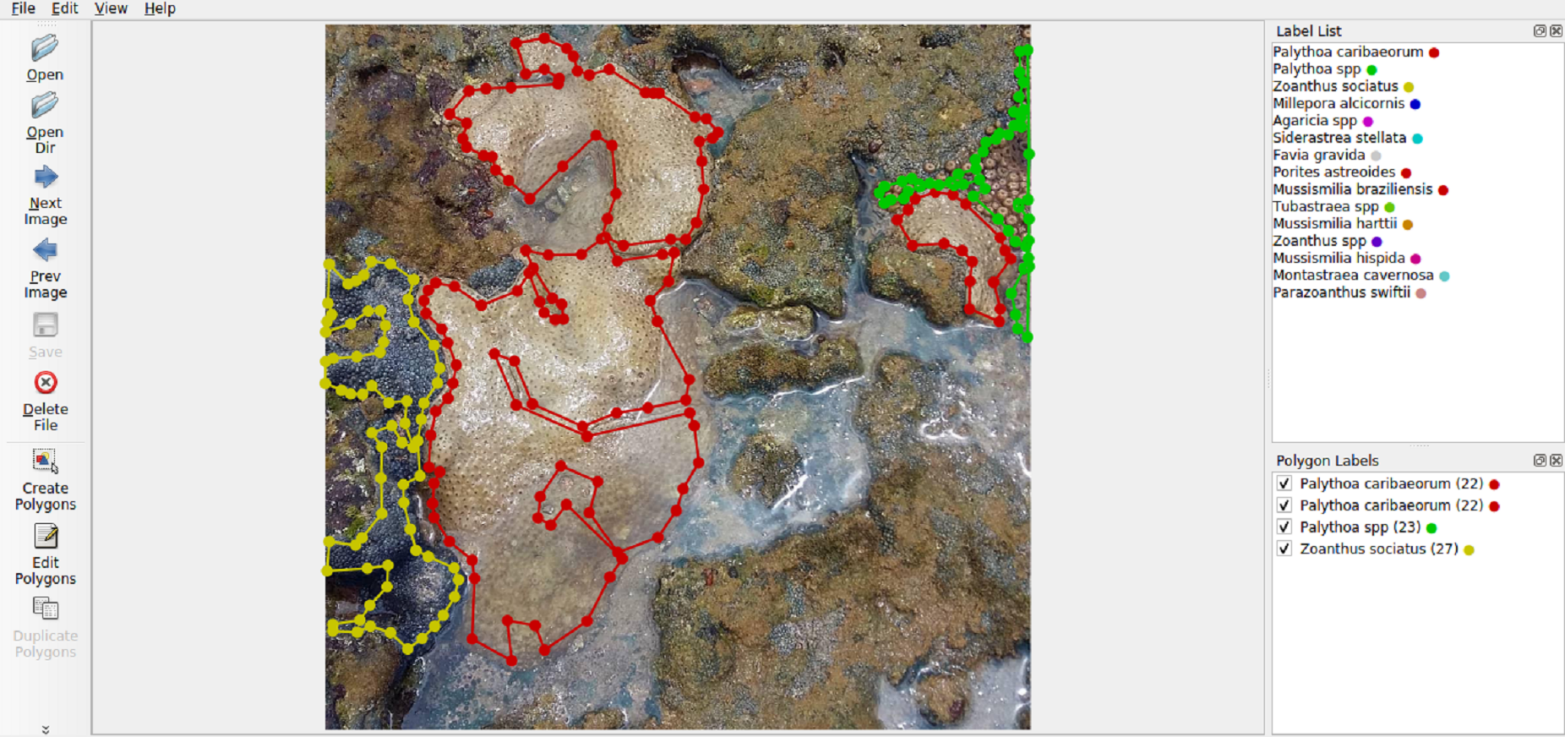
Labelme interface.

The selection of Labelme (Fig. 5) was based on its user-friendly interface for generating polygonal annotations, as well as its capability to export annotations in formats like VOC-format and COCO-format, which are suitable for training machine learning models.

**Figure 6:**
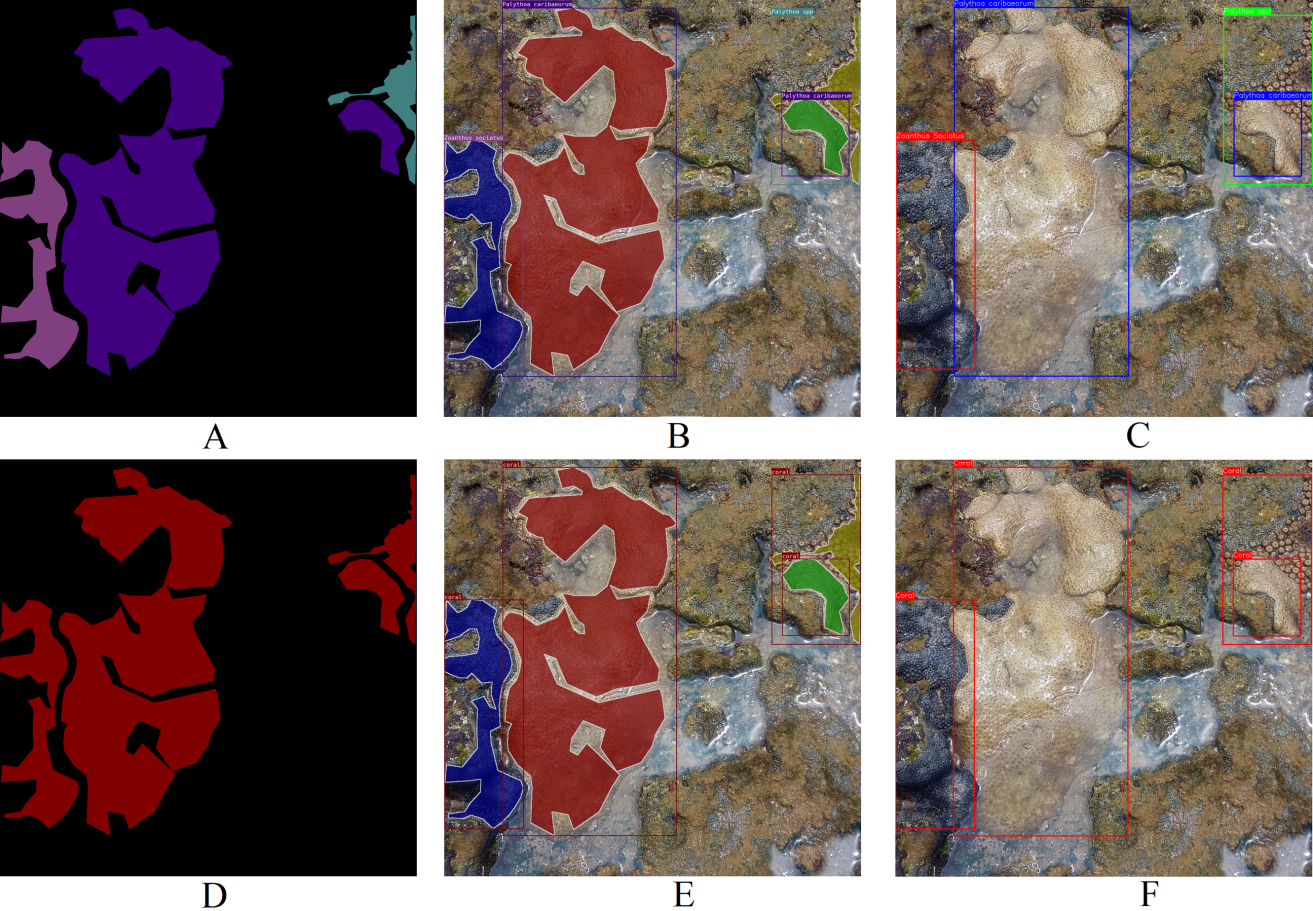
Examples of potential computer vision tasks using Fig. 4A. (A) Categorical semantic segmentation, (B) categorical instance segmentation, (C) categorical object detection, (D) binary semantic segmentation, (E) binary instance segmentation, (F) binary object detection (Furtado, 2022).

Differing from other datasets such as MLC (Beijbom et al., 2012), PLC (Beijbom et al., 2015), Rosenstiel School of Marine and Atmospheric Sciences (RSMAS) dataset (Shihavuddin, 2017), and EILAT dataset (Shihavuddin, 2017), the proposed dataset solely encompasses coral labels. It lacks labels for organisms like algae, sponges, or minerals such as sand within the coral reef ecosystem. These dual labels depicted in Fig. 4 render this dataset a formidable benchmark for key computer vision challenges, such as categorical semantic segmentation (Fig. 6A), binary semantic segmentation (Fig. 6D), categorical instance segmentation (Fig. 6B), binary instance segmentation (Fig. 6E), categorical object detection (Fig. 6C), and binary object detection (Fig. 6F).

A challenge arising from the utilization of a public coral dataset pertains to the necessity for a clear demarcation between training, validation, and testing folds. This predicament impedes result reproducibility and hinders comparisons between different approaches. The creators of the MLC dataset (Beijbom et al. (2012)) opted to partition training and testing data based on the image capture dates. The #DeOlhoNosCorais dataset followed a similar strategy during assembly, given that one of its objectives was to gauge the feasibility of employing machine learning to expedite the analysis of images extracted from social media. This date-based division was chosen to assess models in a real-world context where new images will continually be introduced to the model. The model must demonstrate resilience to technological changes and other variations. Moreover, while cross-validation might maintain a consistent distribution, its results might not be applicable due to situations where a model trained on 2019 images is tested on 2015 images, introducing a random arrangement’s impact on fold difficulty.

To augment the size of the training set, the additional images taken by divers from the research group were incorporated. The Instagram images were allocated to training, validation, and testing sets according to their respective dates. The division rules were as follows:

• Training set: images dated up to 01/01/2019 + additional images from LECOM-UFRN
• Validation set: images dated between 01/01/2019 and 06/30/2019
• Testing set: images dated between 07/01/2019 and 08/24/2021

The extended testing period of two years is due to reduced posts during the Covid-19 pandemic. It is important to emphasize that the testing set emulates potential model encounters in a production environment spanning from 07/01/2019 to 08/24/2021. In reality, the model may confront reposted images and altered image distributions, including shifts in technology and other variables. Table 1 depicts the image distribution across the sets. For the #DeOlhoNosCorais dataset experiments, the validation set was utilized to fine-tune machine learning model hyperparameters. Subsequently, the models were evaluated using the testing set, with training conducted using the combined training and validation sets with the refined hyperparameters.

**Table 1:** Distribution of dataset among training, validation, and testing sets (Furtado, 2022).

	**Training**	**Validation**	**Testing**
Images	889	181	341

Table 2 showcases the proposed dataset’s distribution. Evidently, some classes possess limited samples, while others are absent from specific partitions due to the date-based division. As new images are incorporated into the dataset, this issue will be mitigated.

**Table 2:** #DeOlhoNosCorais dataset (Furtado, 2022).

**Classes**	**Training**	**Validation**	**Testing**
1- *Agaricia* spp	64	5	5
2- *Favia gravida*	57	6	1
3- *Madracis decactis*	6	1	3
4- *Meandrina braziliensis*	0	0	4
5- *Millepora alcicornis*	114	20	33
6- *Millepora braziliensis*	0	1	4
7- *Montastraea cavernosa*	250	29	34
8- *Mussismilia braziliensis*	7	8	6
9- *Mussismilia harttii*	13	8	19
10- *Mussismilia hispida*	36	39	78
11- *Mussismilia leptophylla*	0	1	1
12- *Palythoa caribaeorum*	85	21	59
13- *Palythoa* spp	0	3	8
14- *Parazoanthus swiftii*	2	0	0
15- *Porites astreoides*	50	8	9
16- *Porites branneri*	2	1	2
17- *Scolymia wellsi*	1	7	11
18- *Siderastrea stellata*	168	17	27
19- *Tubastraea* spp	20	4	22
20- *Zoanthus sociatus*	10	1	9
21- *Zoanthus* spp	4	1	6
**Total**	**889**	**181**	**341**

## Experimental Protocol

This section delineates the experimental protocol and metrics adopted for our baseline experiments encompassing both classification and semantic segmentation. In the context of classification tasks, we formulated two distinct experiments. The first experiment involved utilizing the full image to assess the machine learning model’s performance in the face of unaltered original images and associated noise, as illustrated in Fig. 3. Additionally, we employed the Local Interpretable Model-Agnostic Explanations (LIME) algorithm to evaluate the models’ predictions on three testing set images, offering insights into the interpretation of classification outcomes. This experimental approach aimed to simulate real-world scenarios and gauge the models’ efficacy in image classification.

The second experiment focused on leveraging segmentation maps to extract sub-images from the full images. Here, our objective was to compare model outcomes with reduced noise, concentration on coral elements within images, and an augmented dataset volume for training. This experimental setup aligns with earlier endeavors, similar to methodologies applied to the MLC, EILAT, and RSMAS datasets, where coral-centered images were employed for training machine learning models. Shifting our focus to semantic segmentation, we adopted binary labels (coral/not coral). This approach underscored the potential of harnessing machine learning for precise coral localization within entire images. This not only highlighted the potential for improved data clarity but also established a foundation for future explorations, where the potential for localizing and categorically classifying coral could be further investigated. Some portions of this text were previously published as part of a master’s thesis (Furtado, 2022).

### Full image classification

As evident from Table 2, the proposed dataset exhibits substantial class imbalance. To facilitate the classification experiment, a sub-sample of the dataset was chosen, ensuring that each class contained at least 50 images for training. The selected classes and their distribution for the full image classification task are detailed in Table 3.

**Table 3:** Distribution of the sub-sample used for the full image classification experiments (Furtado, 2022).

**Classes**	**Training**	**Validation**	**Testing**
1- *Agaricia* spp	64	5	5
2- *Favia gravida*	57	6	1
3- *Millepora alcicornis*	114	20	33
4- *Montastraea cavernosa*	250	29	34
5- *Palythoa caribaeorum*	85	21	59
6- *Porites astreoides*	50	8	9
7- *Siderastrea stellata*	168	17	27
**Total**	**788**	**106**	**168**

Given the recent surge in popularity and remarkable achievements of deep learning models, we employed two Convolutional Neural Networks (CNNs) for our experiments. The first CNN utilized the EfficientNetB7 (Tan & Le, 2019) for feature extraction and Logistic Regression for classification. A similar model was employed in the work by Chen et al. (2021). In this configuration, the CNN’s dense layers were omitted, and the convolutional layers were initialized with pre-trained weights from the ImageNet dataset (Deng et al., 2009).

In the second scenario, a CNN was trained on the target dataset for feature extraction and classification. The ResNet101 (He et al., 2015) yielded the best results in this experiment. ResNet also exhibited superior performance in prior works such as King, Bhandarkar & Hopkinson (2018), Gómez-Ríos et al. (2019), and Raphael et al. (2020). Two distinct initializations were compared: ImageNet and PLC. In the first case, ResNet101 was initialized solely with ImageNet weights. In the second case, the network was initialized with ImageNet weights and subsequently fine-tuned using the PLC dataset. Further details about the subset of the PLC dataset utilized can be found in Appendix A. Ultimately, both CNNs were trained on the target dataset.

Table 4 presents the training parameters used for the two ResNet101 models in the full image classification task. For both models, a batch size of 32 and dropout (Srivastava et al., 2014) in the dense layer of 0.2 were employed. The training process comprised two steps: i) initialization of the dense layers and ii) training of all layers. For the model (EfficientNetB7 + LR), the Logistic Regression (LR) was trained with the parameter *C* = 1.

**Table 4:** Training parameters for the ResNet101 model in the full image classification task.

			**SGD**		
Model	Training steps	Epoch	Learning rate	*Momentum*	Exponential decay
ResNet101 (ImageNet)	Dense layer	100	10^−4^	0.9	0.005
	Full training	32	10^−5^	0.9	0.020
ResNet101 (PLC)	Dense layer	100	5 × 10^−5^	0.9	0.005
	Full training	48	5 × 10^−6^	0.9	0.020

Following training, the classification protocol includes a step involving analysis using the LIME (Local Interpretable Model-Agnostic Explanations) technique (Ribeiro, Singh & Guestrin, 2016). This algorithm aids in interpreting the results, revealing regions within the image that hold greater importance for the classification outcomes.

In terms of metrics, given the imbalanced nature of the problem, we report the Matthews Correlation Coefficient (MCC) (Eq. 1), F1-score, and Accuracy. These metrics, along with others such as Precision and Recall, can be derived from the counts of True Positives (TP), True Negatives (TN), False Positives (FP), and False Negatives (FN) generated by the classifier.

(1) \begin{eqnarray*}\text{MCC}= \frac{TP\times TN-FP\times FN}{\sqrt{(TP+FP)(TP+FN)(TN+FP)(TN+FN)}} \end{eqnarray*}

The MCC incorporates TP, FP, FP, and FN, yielding a high value (close to 1) when all classes are predicted correctly, even if one or more classes are disproportionately under-represented or over-represented.

### Sub-images classification

**Figure 7:**
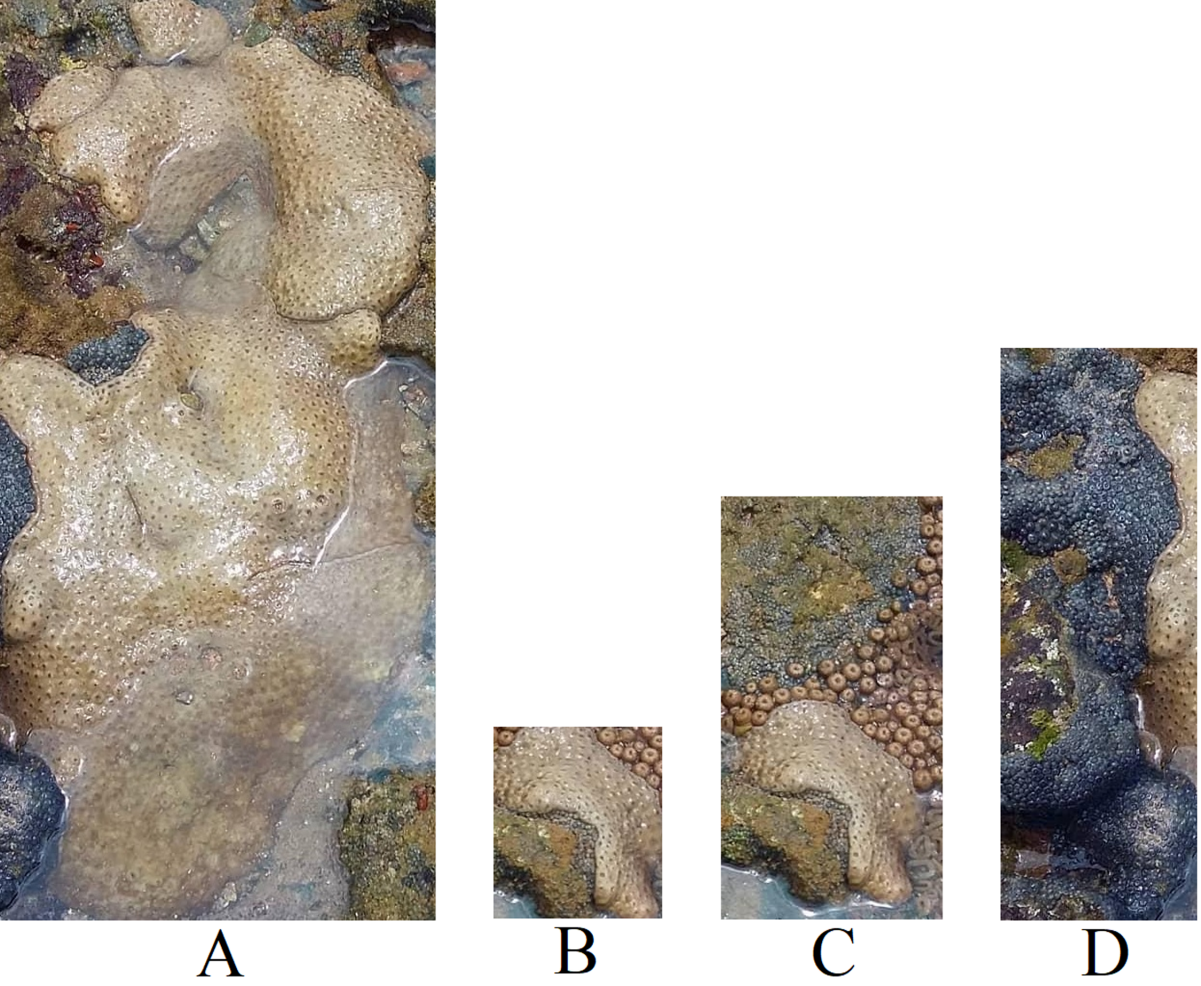
Patches extracted from Fig. 4A. (A) *Palythoa caribaeorum*, (B) *Palythoa caribaeorum* (C) *Palythoa* spp (D) *Zoanthus sociatus* (Furtado, 2022).

To enhance the dataset’s size and explore the impact of noise in the images, as illustrated in Fig. 3, we conducted an experiment using sub-images extracted from the full images. The patches were obtained using bounding boxes generated around the corals, as depicted in Fig. 6C. Figure 7 displays the patches extracted from Fig. 4A, highlighting how the patches mitigate the noise present in the full image.

To enable the classification experiment, we exclusively utilized patches containing more than 50,176 (224 × 224) pixels to ensure high-quality patches and match the input size required by the models used. We maintained the rule of selecting only classes with a minimum of 50 images for training. Table 5 presents the selected classes for the sub-image classification task and their respective distribution. Notably, the number of images increased by 68% compared to the full image dataset (Table 3).

**Table 5:** Distribution of the base used for the sub-images classification task (Furtado, 2022).

**Classes**	**Training**	**Validation**	**Testing**
1- *Agaricia* spp	85	12	9
2- *Favia gravida*	79	6	4
3- *Millepora alcicornis*	137	27	40
4- *Montastraea cavernosa*	303	31	51
5- *Palythoa caribaeorum*	249	37	92
6- *Porites astreoides*	58	16	12
7- *Siderastrea stellata*	432	11	24
8 - *Zoanthus sociatus*	51	3	15
**Total**	**1,394**	**143**	**247**

The same models and methodology used for full image classification were employed, with one alteration: the EfficientNetB7 was replaced with the EfficientNetB0 due to the input size. Table 6 outlines the training parameters for the two ResNet101 models.

**Table 6:** Training parameters for the ResNet101 model for the sub-images classification task (Furtado, 2022).

			**SGD**		
Model	Training steps	Epoch	Learning rate	*Momentum*	Exponential decay
ResNet101 (ImageNet)	Dense layer	100	10^−4^	0.9	0.005
	Full training	12	10^−5^	0.9	0.020
ResNet101 (PLC)	Dense layer	100	5 × 10^−5^	0.9	0.005
	Full training	40	5 × 10^−6^	0.9	0.020

### Binary semantic segmentation

For the semantic segmentation, to utilize the complete dataset outlined in Table 1, we converted the labels into binary form (coral/not coral) to address the class imbalance issue observed in the classification experiments. Table 7 presents the distribution of pixel density among the training, validation, and testing sets, along with the total count of labeled pixels.

**Table 7:** Distribution of pixel density among training, validation, and testing sets.

	**Non-Coral (Background)**	**Coral**	**Total labeled pixels**
Training	70.4%	29.6%	6,643 M
Validation	63.6%	36.4%	164 M
Testing	68.5%	31.5%	275 M

We utilized a modified version of the well-known U-Net architecture, referred to as U-Net Pix2Pix (TensorFlow, 2020). This variant employs MobileNetV2 (Sandler et al., 2018) as the encoder and Pix2Pix (Isola et al., 2016) as the decoder. The model was trained using an Adam optimizer (Kingma & Ba, 2017) with a learning rate of 10^−3^, a batch size of 32, and 20 epochs. In the context of semantic segmentation, the common evaluation metric is the IoU (Intersection over Union). The IoU measures the overlap between the predicted segmentation and the ground truth, divided by the combined area of both. For binary or multi-class segmentation, the mean IoU for an image is computed by averaging the IoU of each individual classes.

## Experimental results

This section presents the outcomes and discussions related to both classification and binary semantic segmentation tasks.

### Full image classification

**Figure 8:**
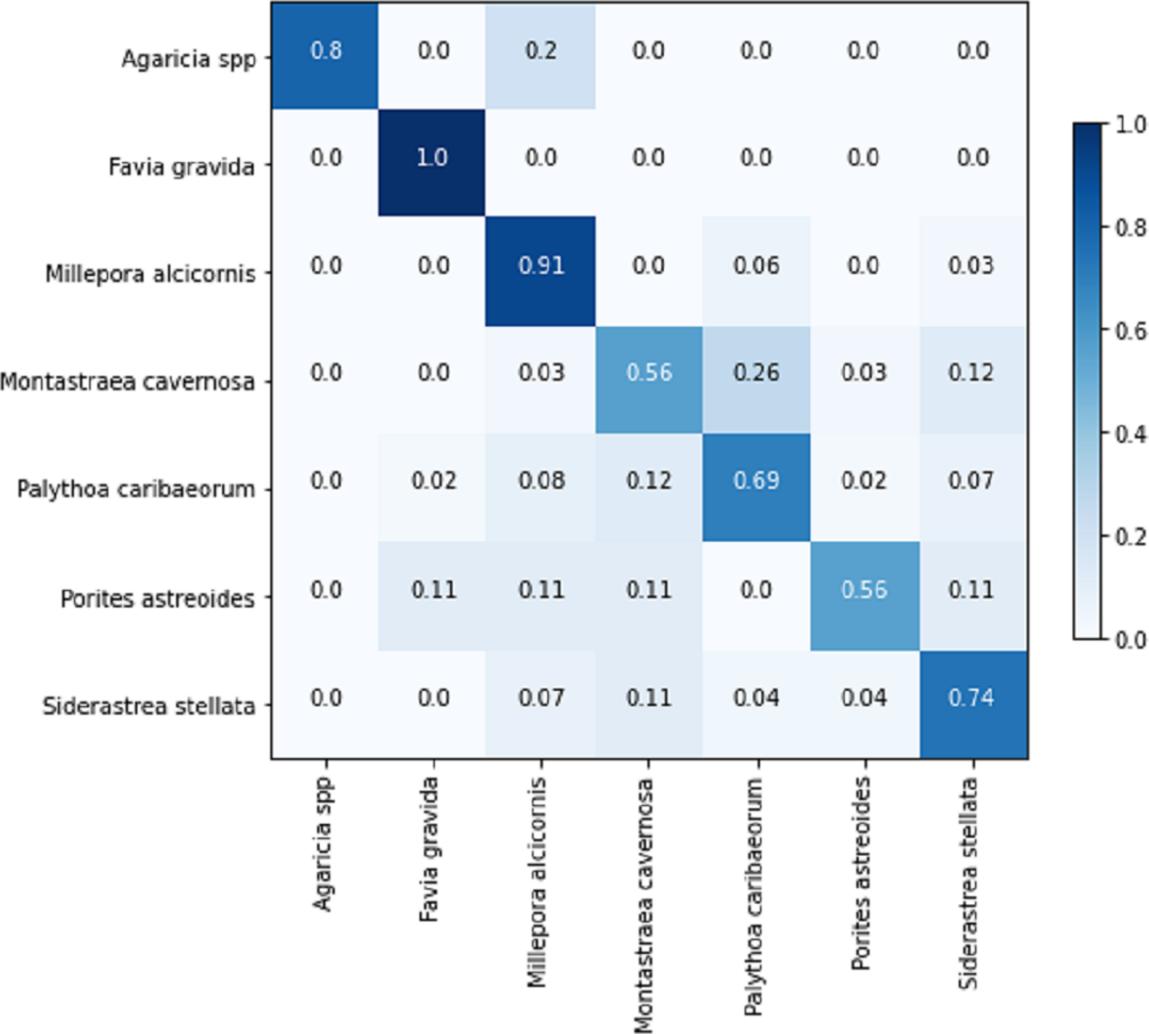
Confusion matrix for the EfficientNetB7 + LR model in the full image classification task on the testing set.

Table 8 displays the achieved metrics for the full image classification task in both validation and testing sets. Notably, the most favorable results were obtained from the EfficientNetB7+LR model. The model trained using the PLC dataset did not lead to an improvement in the performance of the ResNet101. Figure 8 illustrates the confusion matrix for the EfficientNetB7+LR model on the testing set. The model correctly predicted the only image of the class *Favia gravida* in the testing set and achieved an accuracy of 0.9 for the class *Millepora alcicornis*. However, relatively lower performance was observed for the classes *Montastraea cavernosa* and *Porites astreoides*, with accuracies of 0.56.

**Table 8:** Results for the classification task for the validation and testing sets.

	**Accuracy**		**F1**		**MCC**	
	Val.	Testing	Val.	Testing	Val.	Testing
EfficientNetB7 + LR	**0.82**	**0.71**	**0.80**	**0.69**	**0.78**	**0.63**
ResNet101 (ImageNet)	0.73	0.59	0.63	0.53	0.67	0.48
ResNet101 (PLC)	0.67	0.58	0.62	0.51	0.60	0.47

**Notes.**

Bold text represents the best result in each respective column.

**Figure 9:**
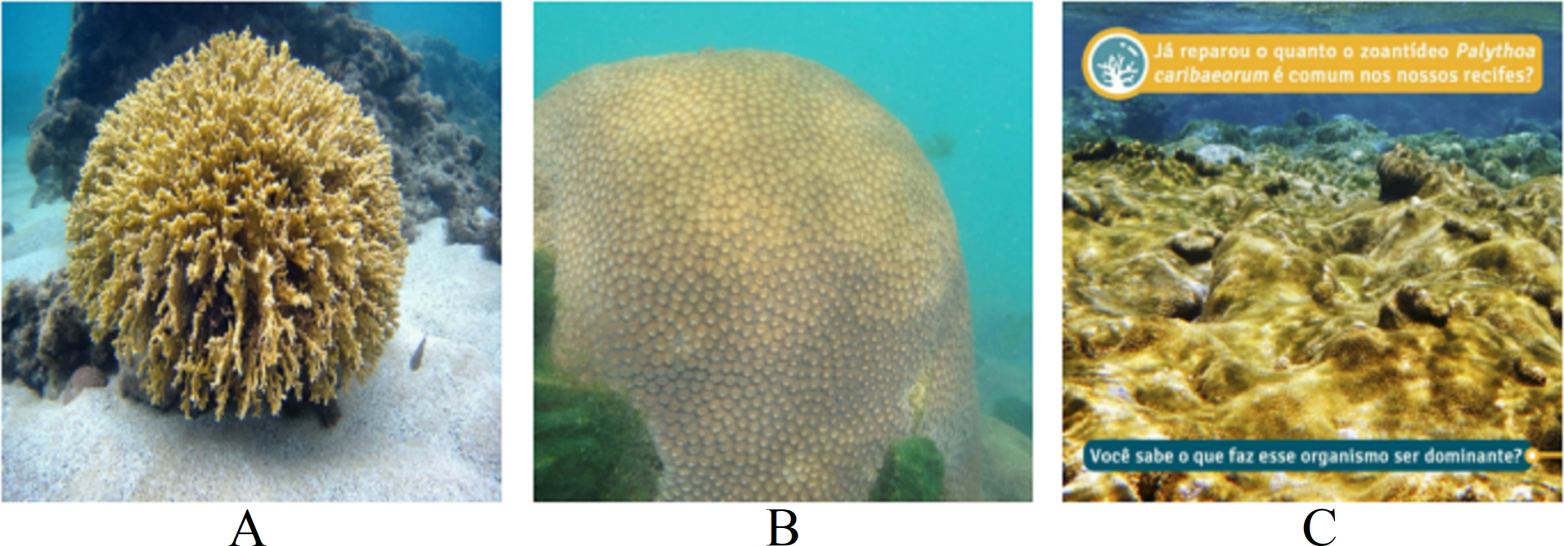
Images extracted from the testing set for the LIME’s analysis. Classes: (A) *Millepora alcicornis*, (B) *Montastraea cavernosa*, (C) *Palythoa caribaeorum*. Text: Have you noticed how common the zoanthid *Palythoa caribaeorum* is in our reefs? Do you know what makes this organism dominant? (Furtado, 2022).

To gain deeper insights into the model’s performance, we chose three images from the testing set for conducting LIME’s analysis (Fig. 9). The results of the models for each image are summarized in Table 9. For Fig. 9A, all classifiers correctly predicted the top class with high probability. In the case of Fig. 9B, while all models misclassified the input, the EfficientNetB7+LR model managed to identify the correct class as its second-best prediction. However, for the last image (Fig. 9C) containing textual information, none of the models correctly classified the input. Two models identified the correct class as their second-best prediction, but with notably low probabilities.

**Figure 10:**
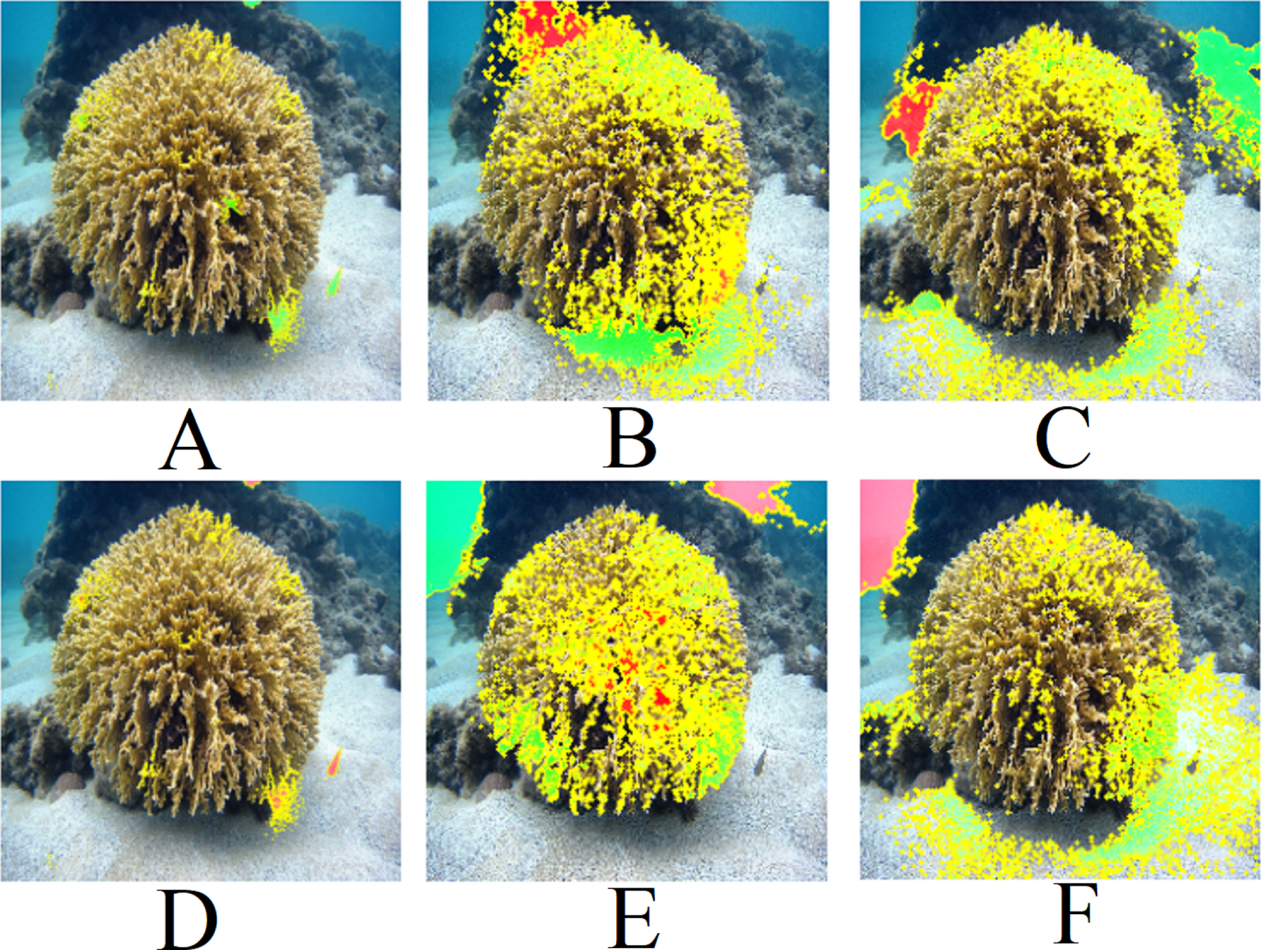
LIME results for Fig. 9A: (A) Top 1 for EfficientNetB7+LR, (B) Top 1 for ResNet101, (C) Top 1 for ResNet (PLC), (D) Top 2 for EfficientNetB7+LR, (E) Top 2 for ResNet101 (ImageNet), (F) Top 2 for ResNet (PLC).

Figure 10 presents the LIME results for Top 1 and Top 2 of the three models shown in Fig. 9A. The LIME algorithm labels the pixels of the image in green and red to indicate positive and negative influences, respectively. Those pixels, red and green, are always surrounded by yellow ones. Therefore, a single green or red pixel is always bordered by yellow. This is why the yellow color dominates the image. As we can observe, the models generally focus on the central coral in the image. The EfficientNetB7+LR model also interacts with a fish and a shadow within the image. However, models based on ResNet101 interact much more with other regions of the image, such as sand and the background. This may explain the lower performance of these methods in the classification tasks.

**Table 9:** Probabilities predicted by the models for Fig. 9. Highest probability class (class 1), second-highest probability class (class 2).

	**Top 1**		**Top 2**	
Input —model	Class	Prob.	Class	Prob.
Fig. 9A —EfficientNetB7 + LR	***Millepora alcicornis***	0.990	*Palythoa caribaeorum*	0.010
Fig. 9A —ResNet101 (ImageNet)	***Millepora alcicornis***	0.990	*Porites astreoides*	0.010
Fig. 9A —ResNet101 (PLC)	***Millepora alcicornis***	0.999	*Favia gravida*	0.001
Fig. 9B —EfficientNetB7 + LR	*Siderastrea stellata*	0.540	***Montastraea Cavernosa***	0.450
Fig. 9B —ResNet101 (ImageNet)	*Siderastrea stellata*	0.970	*Palythoa caribaeorum*	0.020
Fig. 9B —ResNet101 (PLC)	*Siderastrea stellata*	0.999	***Montastraea Cavernosa***	0.001
Fig. 9C —EfficientNetB7 + LR	*Siderastrea stellata*	0.710	***Palythoa caribaeorum***	0.120
Fig. 9C —ResNet101 (ImageNet)	*Porites astreoides*	0.520	*Siderastrea stellata*	0.220
Fig. 9C —ResNet101 (PLC)	*Porites astreoides*	0.550	***Palythoa caribaeorum***	0.220

**Notes.**

Bold text indicates the correct class for the corresponding Figure.

**Figure 11:**
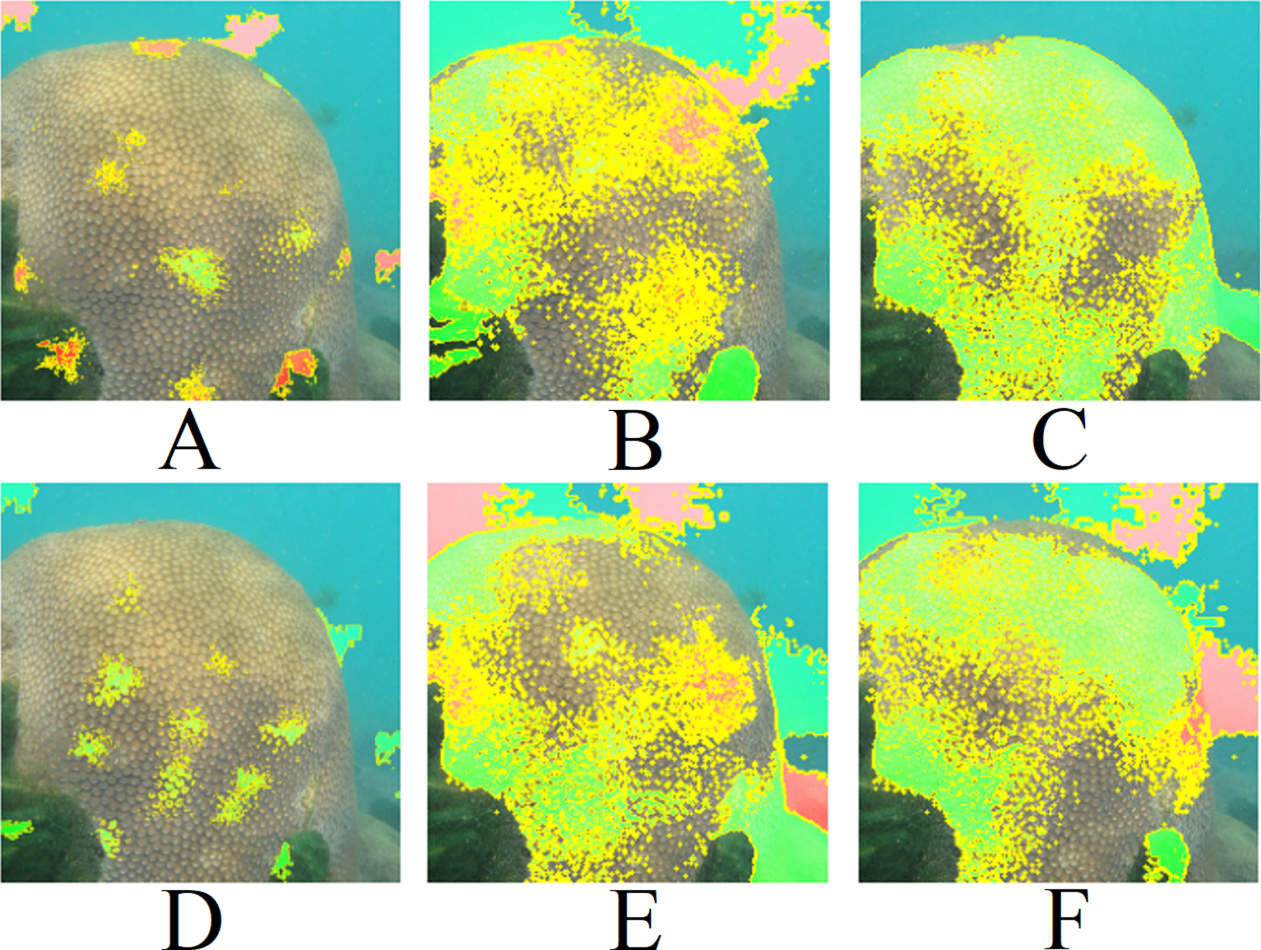
LIME results for Fig. 9B: (A) Top 1 for EfficientNetB7+LR, (B) Top 1 for ResNet101, (C) Top 1 for ResNet (PLC), (D) Top 2 for EfficientNetB7+LR, (E) Top 2 for ResNet101 (ImageNet), (F) Top 2 for ResNet (PLC).

Figure 11 presents the LIME results for Fig. 9B. Similar to Fig. 9A, the models focus on the central region of the coral. However, the ResNet101 models consider a considerable amount of the background.

**Figure 12:**
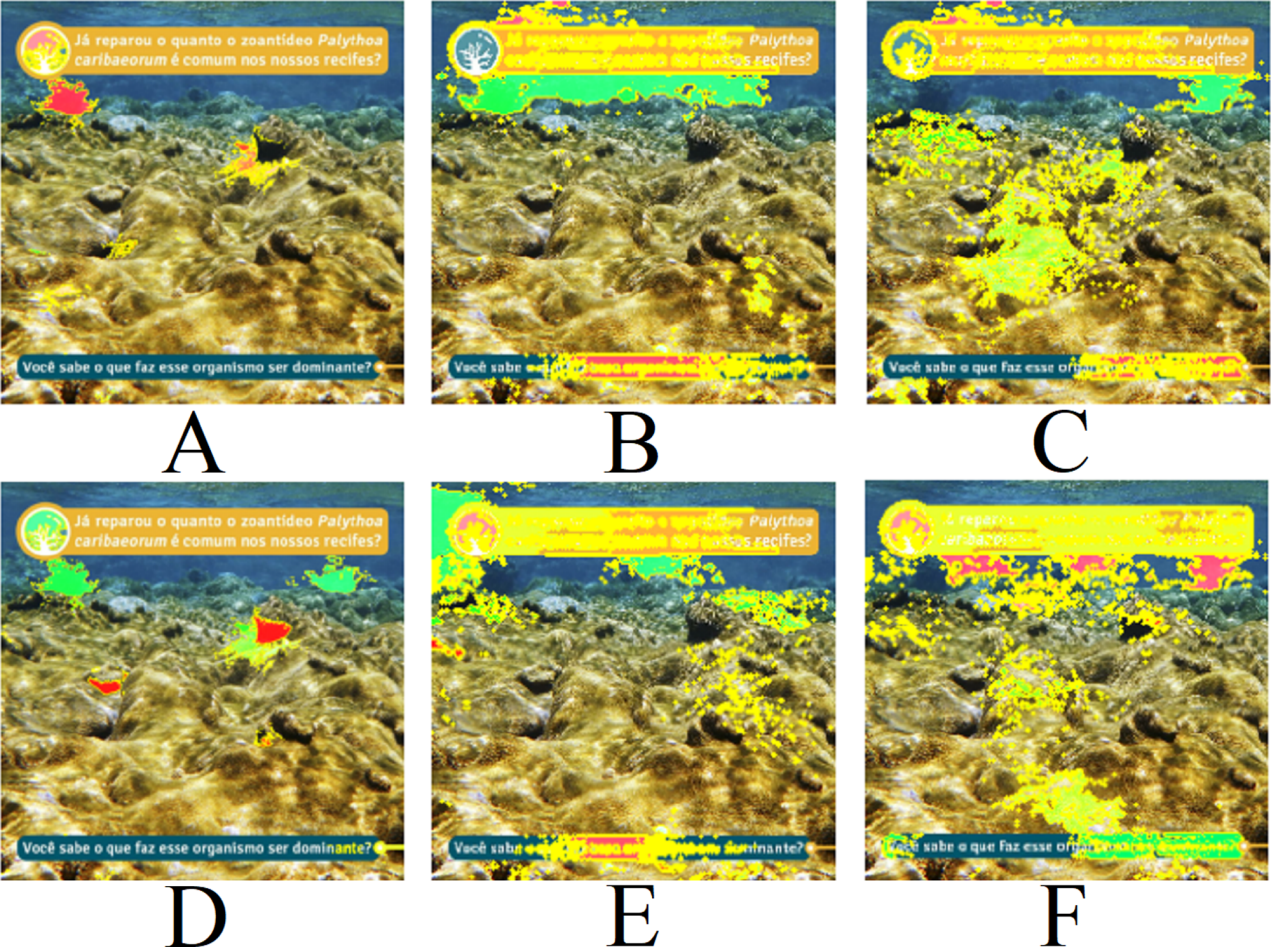
LIME results for Fig. 9C: (A) Top 1 for EfficientNetB7+LR, (B) Top 1 for ResNet101, (C) Top 1 for ResNet (PLC), (D) Top 2 for EfficientNetB7+LR, (E) Top 2 for ResNet101 (ImageNet), (F) Top 2 for ResNet (PLC)). Text: Have you noticed how common the zoanthid *Palythoa caribaeorum* is in our reefs? Do you know what makes this organism dominant?.

Finally, Fig. 12 presents the LIME results for Fig. 9C. An interesting aspect of this image is the absence of centered coral and the presence of text. It is noted that all three models interact with the presence of text in the image, evaluating it positively or negatively. Therefore, the predictions for the image are unreliable due to interactions with other artifacts in the image. Consequently, removing text, watermarks, and noise is necessary for achieving a more reliable model prediction.

### Sub-images classification

The results for the sub-image classification task are presented in Table 10. The results demonstrate the superiority of EfficientNet+LR over the ResNet models in both the validation and testing sets, mirroring the results obtained when using the full image. Another noteworthy observation is the performance disparity among the various ResNet models. ResNet101 (ImageNet) outperformed ResNet101 (PLC) on the validation set. However, the performance of ResNet101 (ImageNet) significantly declined on the testing set, whereas ResNet101 (PLC) consistently maintained its performance across sets. Consequently, the utilization of the PLC dataset contributed to the enhancement of the model’s generalization.

**Figure 13:**
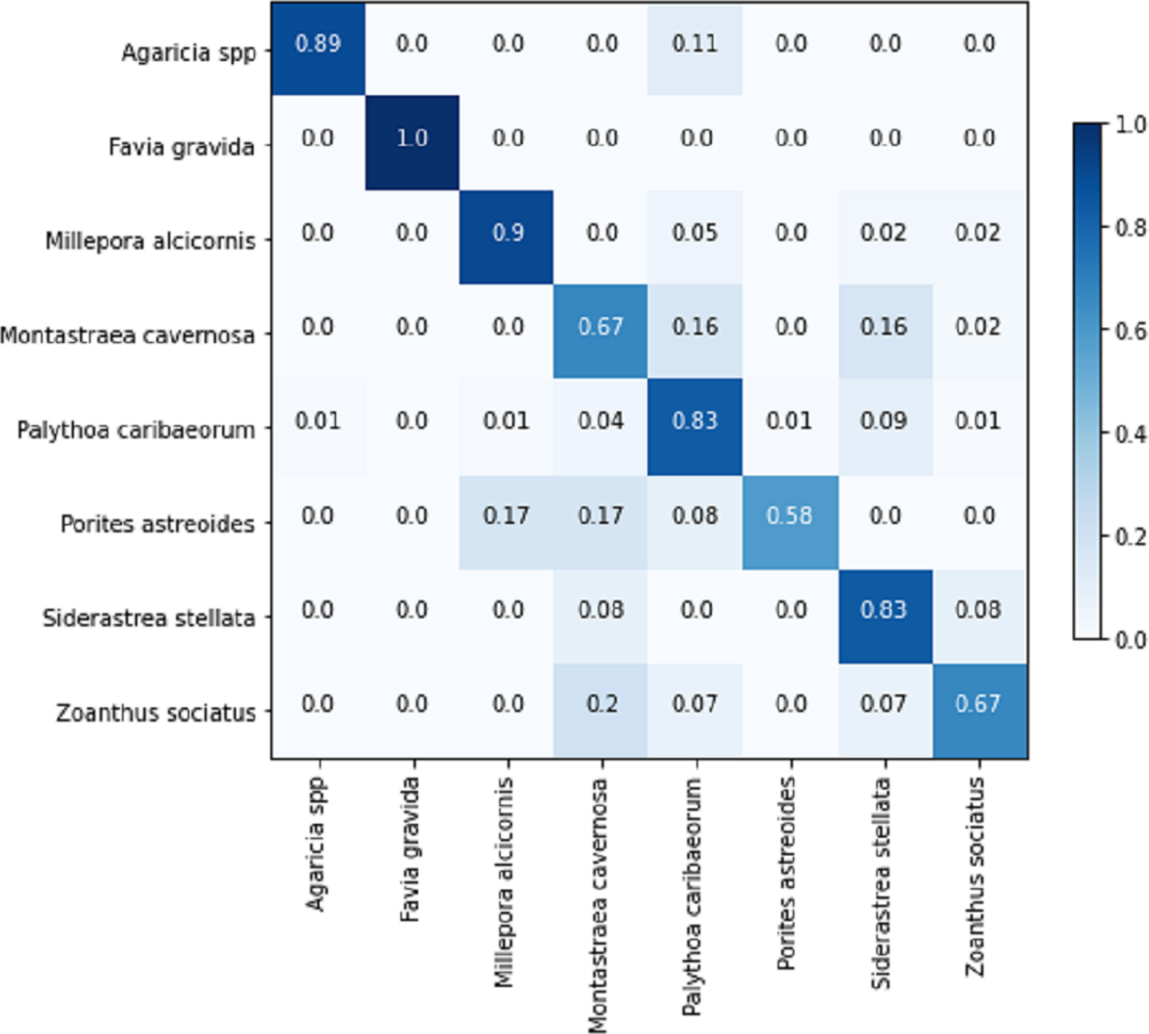
EfficientNetB0 + LR confusion matrix for the sub-images classification task on the testing set.

The confusion matrix for the sub-image classification task using the EfficientNetB0+LR model on the testing set is illustrated in Fig. 13. In comparison to the full-image task, the performance on the validation set exhibited similarity, considering the inclusion of an additional class in the sub-image task. However, upon comparing the results of the testing set, the sub-image task demonstrated an approximately 10% performance improvement. This illustrates that, in certain cases, the additional information within the image adversely affects the model’s predictions. Another noteworthy aspect is that the increased number of images also contributes to the models’ improved generalization.

**Table 10:** Results for the sub-images classification task for the validation and testing sets.

	**Accuracy**		**F1**		**MCC**	
	Val.	Testing	Val.	Testing	Val.	Testing
EfficientNetB0 + LR	**0.80**	**0.79**	**0.76**	**0.80**	**0.77**	**0.73**
ResNet101 (ImageNet)	0.78	0.71	0.75	0.70	0.73	0.62
ResNet101 (PLC)	0.73	0.74	0.71	0.73	0.69	0.66

**Notes.**

Bold text represents the best result in each respective column.

### Binary semantic segmentation

The results for binary semantic segmentation using the U-net (Pix2Pix) model are presented in Table 11. The model achieved comparable results in both sets, indicating strong generalization.

**Figure 14:**
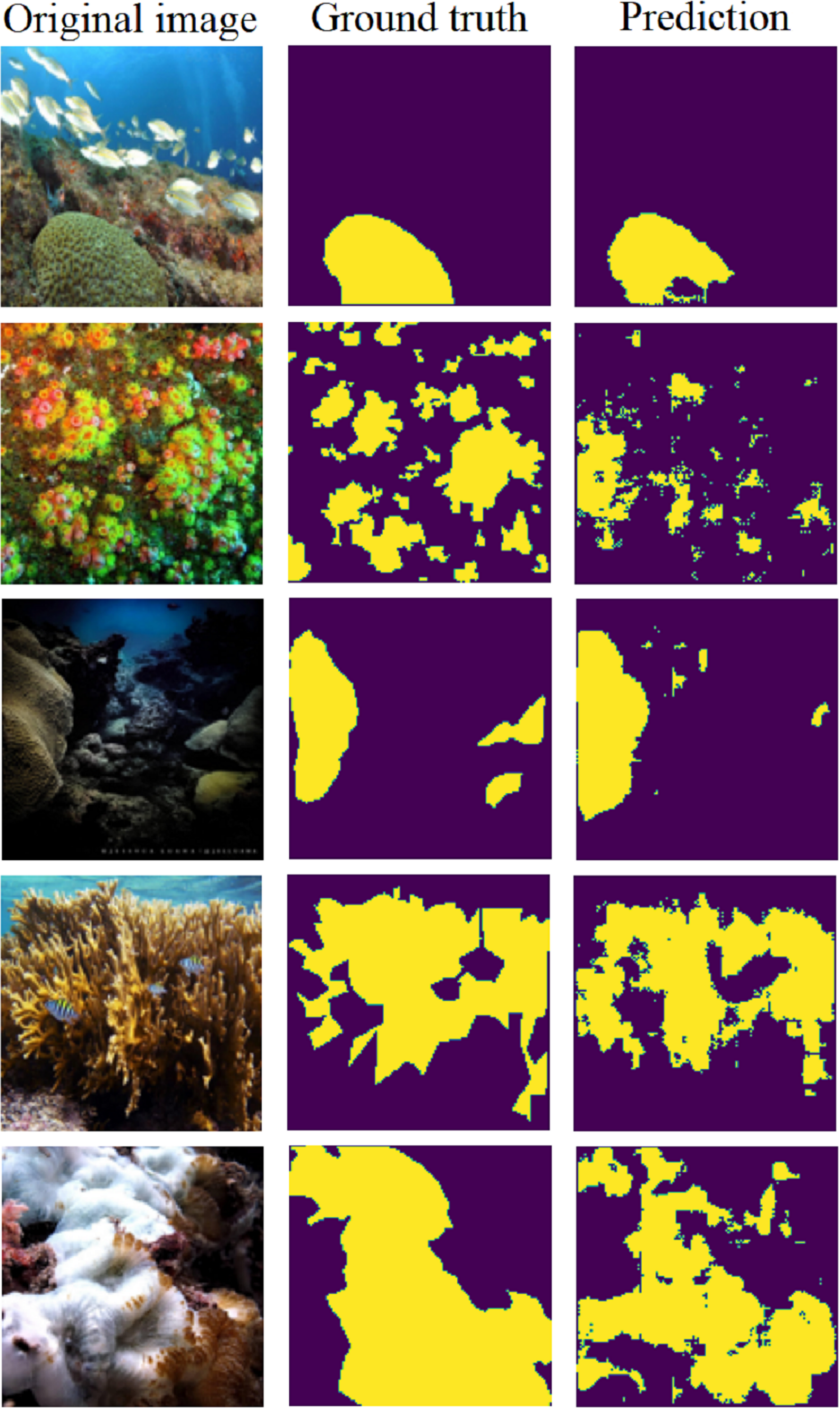
Five examples of predictions using the U-net (Pix2Pix) on the testing set (Furtado, 2022).

Figure 14 displays five samples chosen from the testing set. It depicts the original image, ground truth, and the predicted segmentation map. These examples illustrate the model’s ability to accurately locate corals in diverse scenarios within the image.

**Table 11:** Results for the binary semantic segmentation task using the U-net (Pix2Pix) for the validation and testing sets (Furtado, 2022).

	**Accuracy per pixel**		**mIoU**	
	Val.	Testing	Val.	Testing
U-net (Pix2Pix)	0.86	0.86	0.74	0.70

## Conclusion

In this paper, we introduced the #DeOlhoNosCorais dataset, consisting of 1,411 images of scleractinian corals, hydrocorals and zoanthids along the Brazilian coast, each accompanied by its corresponding segmentation map. The dataset encompasses 7,082 M labeled pixels, offering potential applications across various tasks and in conjunction with other datasets. We proposed a straightforward methodology to partition the data into training, validation, and testing sets, ensuring fair comparisons. Additionally, we conducted baseline experiments for classification and segmentation tasks, establishing a foundation for future comparisons.

Concerning the classification task, the model leveraging EfficientNetB7 for feature extraction and Logistic Regression for classification outperformed the end-to-end trained ResNet101 in both scenarios: using the full image and sub-images. While achieving accuracy and F1 Score of 0.70 and 0.80 with the full image and sub-images respectively, there remains room for improvement. As a prospective avenue, we suggest employing Generative Adversarial Networks (GANs) to synthetically expand the training dataset. LIME algorithm results indicated that the classification models prioritize corals as significant regions for predictions. Furthermore, the models are susceptible to influences from artifacts introduced by users on social platforms, such as text boxes, potentially leading to erroneous predictions.

The semantic segmentation experiments offer promising avenues for enhancing classification outcomes. By exclusively utilizing regions highlighted by the segmentation process, noise areas like those containing textual information can be mitigated.

## Appendix A

**PLC**

Table A1 illustrates the distribution of the sub-base within the PLC dataset utilized for model training. Out of the 20 available classes, twelve were selected for inclusion. Image patches of size 224 × 224 pixels were extracted, centered on the labeled pixel.

**Table A1:** Distribution of the PLC dataset used for training the ResNet101.

**Classes**	**Training**	**Validation**
1- *Acropora*	5,181	159
2- *Favia*	1,343	74
3- *Favites*	1,124	55
4- *Macroalgae*	22,783	700
5- *Millepora*	3,114	158
6- *Montipora*	12,082	444
7- *Other scleractinians*	21,614	724
8- *Pavona*	2,033	52
9- *Platygyra*	703	44
10- *Pocillopora*	9,756	244
11- *Porites*	11,400	332
12- *Soft Coral*	4,860	144
**Total**	**95,993**	**3,130**

For training purposes, images from the reference set were employed, while the evaluation set with archived labels served as the validation set. Table A2 presents the training parameters for the ResNet101 model on the PLC dataset. A batch size of 32 was employed, along with a dropout rate of 0.2 in the dense layer. The model was pre-trained on the ImageNet dataset.

**Table A2:** Training parameters for the ResNet101 model on the PLC dataset.

		**SGD**		
Training steps	Epoch	Learning rate	*Momentum*	Exponential decay
Dense layer	43	10^−4^	0.9	0.005
Full training	59	10^−5^	0.9	0.020

## Additional Information and Declarations

### Data Availability

The following information was supplied regarding data availability: The data is available at Zenodo: Daniel Pimenta Furtado, Edson Aparecido Vieira Filho, Wildna Fernandes do Nascimento, Kelly Yumi Inagaki, Jessica Bleuel, Marco Antonio Zanata Alves, Guilherme Ortigara Longo, & Luiz Eduardo Soares de Oliveira. (2022). #DeOlhoNosCorais: a polygonal annotated dataset to optimize coral monitoring (Version v1) (Data set). Zenodo. https://doi.org/10.5281/zenodo.7338208
The code is available at Github and Zenodo: - https://github.com/danielpimentaf/DeOlhoNosCorais
- Daniel Pimenta Furtado, Marco Antonio Zanata Alves, & Luiz Eduardo Soares de Oliveira. (2023). #DeOlhoNosCorais: a polygonal annotated dataset to optimize coral monitoring (GitHub) (v.1.2). Zenodo. https://doi.org/10.5281/zenodo.7897923.
